# Cytokine dynamics and targeted immunotherapies in autoimmune encephalitis

**DOI:** 10.1093/braincomms/fcac196

**Published:** 2022-08-20

**Authors:** Nicolás Lundahl Ciano-Petersen, Sergio Muñiz-Castrillo, Cristina Birzu, Alberto Vogrig, Antonio Farina, Macarena Villagrán-García, Bastien Joubert, Dimitri Psimaras, Jérôme Honnorat

**Affiliations:** French Reference Center on Paraneoplastic Neurological Syndromes and Autoimmune Encephalitis, Hospices Civils de Lyon, Hôpital Neurologique, Bron, France; SynatAc Team, Institute MeLiS, INSERM U1314/CNRS UMR 5284, Université de Lyon, Université Claude Bernard Lyon 1, Lyon, France; Neuroimmunology and Neuroinflammation group. Biomedical Research Institute of Málaga (IBIMA), Málaga, Spain; Red Andaluza de Investigación Clínica y Traslacional en Neurología (Neuro-RECA). Hospital Regional Universitario de Málaga, Málaga, Spain; French Reference Center on Paraneoplastic Neurological Syndromes and Autoimmune Encephalitis, Hospices Civils de Lyon, Hôpital Neurologique, Bron, France; SynatAc Team, Institute MeLiS, INSERM U1314/CNRS UMR 5284, Université de Lyon, Université Claude Bernard Lyon 1, Lyon, France; Service de Neurologie 2-Mazarin, Centre de Recherche de l’Institut du Cerveau et de la Moelle Epinière, Groupe Hospitalier Pitie-Salpetrière et Université Pierre et Marie Curie-Paris 6, AP-HP, Paris, France; French Reference Center on Paraneoplastic Neurological Syndromes and Autoimmune Encephalitis, Hospices Civils de Lyon, Hôpital Neurologique, Bron, France; SynatAc Team, Institute MeLiS, INSERM U1314/CNRS UMR 5284, Université de Lyon, Université Claude Bernard Lyon 1, Lyon, France; French Reference Center on Paraneoplastic Neurological Syndromes and Autoimmune Encephalitis, Hospices Civils de Lyon, Hôpital Neurologique, Bron, France; SynatAc Team, Institute MeLiS, INSERM U1314/CNRS UMR 5284, Université de Lyon, Université Claude Bernard Lyon 1, Lyon, France; French Reference Center on Paraneoplastic Neurological Syndromes and Autoimmune Encephalitis, Hospices Civils de Lyon, Hôpital Neurologique, Bron, France; SynatAc Team, Institute MeLiS, INSERM U1314/CNRS UMR 5284, Université de Lyon, Université Claude Bernard Lyon 1, Lyon, France; French Reference Center on Paraneoplastic Neurological Syndromes and Autoimmune Encephalitis, Hospices Civils de Lyon, Hôpital Neurologique, Bron, France; SynatAc Team, Institute MeLiS, INSERM U1314/CNRS UMR 5284, Université de Lyon, Université Claude Bernard Lyon 1, Lyon, France; Service de Neurologie 2-Mazarin, Centre de Recherche de l’Institut du Cerveau et de la Moelle Epinière, Groupe Hospitalier Pitie-Salpetrière et Université Pierre et Marie Curie-Paris 6, AP-HP, Paris, France; French Reference Center on Paraneoplastic Neurological Syndromes and Autoimmune Encephalitis, Hospices Civils de Lyon, Hôpital Neurologique, Bron, France; SynatAc Team, Institute MeLiS, INSERM U1314/CNRS UMR 5284, Université de Lyon, Université Claude Bernard Lyon 1, Lyon, France

**Keywords:** autoimmune encephalitis, NMDAR, LGI1, cytokines, targeted immunotherapies

## Abstract

Autoimmune encephalitides constitute a diverse group of immune-mediated central nervous system disorders mainly characterized by the presence of antibodies targeting neuronal or glial antigens. Despite the notable contribution of antibody discovery to the understanding of their physiopathology, the specific immune cells and inflammatory mediators involved in autoimmune encephalitis are still poorly defined. However, cytokines have recently emerged as crucial signalling molecules in the pathogenesis of autoimmune encephalitis. Cytokines are biologically active, soluble, low-molecular-weight proteins or glycoproteins involved in a wide variety of physiological functions, including central nervous system development and homeostasis, immune surveillance, as well as proliferation and maturation of immune cells. Since unbalanced cytokine expression is considered a hallmark of many autoimmune central nervous system disorders, their identification and quantification has become an essential element in personalized medicine applied to the field of neuroimmunology. Several studies have explored the cytokine profile of autoimmune encephalitis, but their interpretation and comparison is challenging due to their small sample sizes and extremely high heterogeneity, especially regarding the cytokines analysed, type of sample used, and associated neural antibody. Only the cytokine profile of anti-N-methyl-**D**-aspartate receptor encephalitis has extensively been investigated, with findings suggesting that, although humoral immunity is the main effector, T cells may also be relevant for the development of this disorder. A better understanding of cytokine dynamics governing neuroinflammation might offer the opportunity of developing new therapeutic strategies against specific immune cells, cytokines, antibodies, or intracellular signalling cascades, therefore leading to better outcomes and preventing undesired side effects of the presently used strategies. In this review, we first summarize the current knowledge about the role of cytokines in the pathogenesis of autoimmune encephalitis, combining theoretical analysis with experimental validations, to assess their suitability as clinical biomarkers. Second, we discuss the potential applicability of the novel targeted immunotherapies in autoimmune encephalitis depending on the immunobiology of the associated antibody, their limitations, as well as the main limitations that should be addressed in future studies.

## Teaser

Ciano-Petersen *et al*. extensively review the value of cytokines as clinical biomarkers in autoimmune encephalitis, summarize the current knowledge about their physiopathological role, and additionally discuss in detail the most relevant targeted immunotherapies used in autoimmune encephalitis according to their underlying pathogenesis and associated antibody.

## Graphical Abstract

**Graphical abstract:**
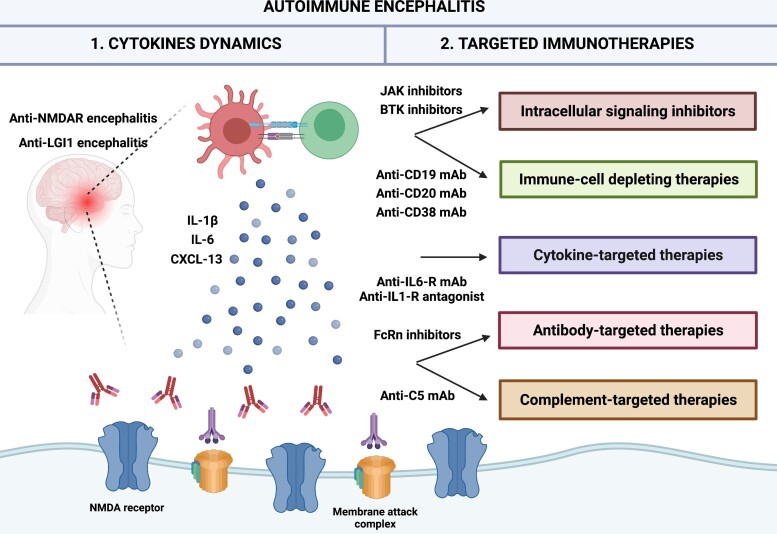

## Introduction

Autoimmune encephalitides (AEs) comprise immune-mediated CNS disorders mainly characterized by the presence of antibodies against neuronal or glial antigens. Some of the most recent advances in the knowledge of AE include a more accurate diagnosis thanks to improved antibody detection, the deciphering of the pathogenic roles of these antibodies, and the different responses to current treatments according to the targeted antigen.^1^ However, the specific immune cells and inflammatory mediators contributing to the development of neuroinflammation have so far been poorly investigated, although cytokines are signalling molecules promising for the understanding of the pathogenesis of AE.

Cytokines constitute a group of small proteins that have a major role in the development of inflammation and immune regulation.^2,3^ Moreover, since an unbalanced cytokine expression is characteristic of many autoimmune CNS disorders, their quantification has become an essential element in neuroimmunology, acquiring a major role as diagnostic, prognostic, and therapeutic biomarkers.^2,4^ Their application in the era of ‘biomarker-guided therapy’ has led to a paradigm shift in the field, providing a wide therapeutic arsenal to modulate the specific inflammatory pathways involved in autoimmune CNS disorders.^5^

In the present article, we provide a theoretical analysis and review experimental validations to evaluate cytokines as clinical biomarkers in AE and summarize the current knowledge about their role in the pathogenesis of these diseases. In addition, we discuss the most relevant targeted immunotherapies in AE according to their underlying pathogenesis and associated antibody, as well as the main limitations that should be addressed in future studies.

### Cytokines and chemokines

Cytokines are biologically active, soluble, low-molecular-weight proteins or glycoproteins involved in a wide range of physiological functions, such as CNS development and homeostasis, immune surveillance, as well as proliferation and maturation of immune cells.^6,7^ Furthermore, they are highly important signalling molecules, not only for immune cells to communicate and coordinate their biological activities but also for neurons and microglia.^8^ Cytokines can be classified according to their ability to attract immune cells into specific organs. Non-chemoattractant cytokines mainly regulate the proliferation and maturation of immune cells and comprise tumour necrosis factors (TNFs), interleukins (ILs), interferons (IFNs), and growth factors such as transforming growth factor (TGF). In contrast, chemotactic cytokines (chemokines) have the ability to attract and guide leukocyte migration into affected organs.^8^ These molecules establish a wide biochemical network that coordinates the interaction between innate and adaptive immune cells and can be further classified according to their role in the recruitment, proliferation, and activation of different subsets of immune cells.^8, 9^

The cytokine microenvironment regulates the proliferation and polarization of precursor immune cells into functional subsets, such as the shift of naïve CD4+ T**-**cells into Th1-, Th2-, Th9-, Th22-, T follicular helper, and Th17-cells. Among these, Th1-, Th2-, and Th17-cells have been reported to have a role in CNS autoimmunity (Table 1). This transformation is driven by the binding of cytokines to their receptors (CKR), leading to the activation of a cascade of downstream signalling molecules, such as Janus kinases (JAKs), and signal transducers and activators of transcription (STAT), which ultimately regulate gene transcription necessary for specialized tasks.^10^ Although some cytokines may bind several CKR, most of them exhibit a higher affinity for specific CKR expressed by certain immune cells, and therefore play a role in particular immune responses.^8^

**Table 1:** Interactions between cytokines and different immune cells involved in immune responses

Immune cells	Function	Attracted by	Induced by	Produce
**Th1-cells**	Regulate cellular immunity, orchestrating expansion of cytotoxic T cells and ASC	CXCL-9–11, CCL3	IFN-**γ**, IL-12	IFN-y, IL-2, TNF-α, CXCL9-11
**Th2-cells**	Regulate humoral immunity, relevant in allergy	CCL1,8,17, 22	IL-4	IL4-6, 13, 25, 31, 33; CCL21
**Th17-cells**	Major role in neuroinflammatory disorders, can further shift towards Th1 or Th2 phenotype	CXCL-13, CCL22	TGF-β, IL-6, IL-23	IL-1β, 6, 8, 17A, 21-23,
**Treg-cells**	Regulate immune responses and immune tolerance	CCL1, CCL22	TGF-β, IL-2	IL-10, IL-35, TGF-β
**B-cells**	Regulate humoral immunity effectors	CXCL-10, CXCL-13	IL-1β, IL-6	IL-5, 10, 12, 14; TNF-β

ASC, antibody-secreting cells CCL, CC chemokine ligand; CXCL, C-X-C motif chemokine; IFN, interferon; IL, interleukin; TGF, transforming growth factor; Th, T-helper cell; TNF, tumour necrosis factor; Treg, regulatory T cells.

The characterization of immune cells based on their cytokine signature and CKR has recently gained interest, as particular T-cell subsets may predominate in certain neuroimmune disorders.^11^ Hence, a better definition of the dynamics of these cells and their cytokine expression could unravel the immunological pathways and mechanisms responsible for the development of AE, which, in turn, is crucial for the identification of candidates for new-targeted immunological therapies.

## Methods

For the evaluation of cytokines as clinical biomarkers in AE, we conducted an exhaustive search of articles published from 1st January 1995 to 15th May 2021 in MEDLINE/Pubmed. The following MeSH terms, free text, and related search terms were included: ‘Autoimmune encephalitis’ AND (‘cytokine’ OR ‘chemokine’ OR ‘interleukin’ OR ‘inflammatory mediator’). The first screening of results was performed by titles and abstracts, and only studies with full text available in English language were included. Additional articles were retrieved by searching through the reference lists of all studies. Only studies analysing cytokines in the CSF and including at least 20 patients with the same type of AE were selected, regardless of the methodology used to quantify cytokine levels. Despite myelin oligodendrocyte glycoprotein antibody-associated disease and neuromyelitis optica spectrum disorders (NMOSD) being also associated with acute disseminated encephalomyelitis and other encephalitic syndromes, they were not included in this study.

### Data availability

Data sharing is not applicable to this article as no new data were created or analyzed in this study.

### The role of cytokine networks in autoimmune encephalitides

Most studies reviewed examined cytokine profiles in AE with immunoassay-based methodologies. Depending on the cytokine analysed and the commercial kit used, cytokines were assessed by either a single plex assay using conventional sandwich ELISA, or by multiplexing strategies with fluorescent bead-based immunoassays that allow the detection of several cytokines in a small amount of sample. However, the limited size of the study populations, as well as the heterogeneity of cytokines assessed, types of samples, and patient characteristics make it difficult to interpret and compare the reported results. For these reasons, in an attempt to homogeneously present these data and ease its understanding, we focused on cytokines analysed in CSF and in a significant number of patients with the same AE, and summarized findings according to the associated neural antibody and immune response involved.

#### Anti-N-methyl-D-aspartate receptor encephalitis

Anti-N-methyl-D-aspartate receptor (NMDAR) encephalitis is an autoimmune disorder characterized by the presence of IgG1 antibodies against NMDAR.^12,13^ Meaningful investigations have been conducted on cultured neurons^12,14^ and *in vivo* models,^15^ demonstrating the pathogenic role of the antibodies that provoke the internalization of NMDAR and disrupt its interaction with Ephrin B2 receptor.^16^ The active role of these antibodies and the expansion of CD19+ B-cells and CD19 + CD138 + plasma cells in CSF suggest that humoral immunity is key to anti-NMDAR encephalitis.^17–20^ However, although histopathological studies found intense infiltration of CD138 + plasma cells in the brain parenchyma and mild infiltrations of B-cells in perivascular spaces, CD3+ T-cells were also identified, reflecting that cellular immunity may play also a role. Nevertheless, there is no evidence of antibody and complement deposition or neuronal loss, which may explain the generally good outcome with immunotherapy.^21, 22^

Several cytokines implicated in B- and T-cell activation have been found in the CSF of patients with anti-NMDAR encephalitis^23^ (Table 2). Among cytokines regulating the B-cell axis, C-X-C motif chemokine (CXCL) 13 is considered the main B-cell recruiter (*via* CXCR5) into the CNS, although it is also involved in Th17/Treg ratio regulation and Th17-B-cell interactions necessary for antibody production.^9^ High intrathecal CXCL-13 levels have been reported in a total of 271 patients with anti-NMDAR encephalitis.^24–29^ Intriguingly, a paired serum-CSF study found higher concentrations in serum than in CSF,^25^ whereas another found increased levels only in the CSF.^24^ The CXCL-13 CSF/serum ratio is considered a measure of chemotactic gradient that may change during the disease course, likely explaining the differences in serum CXCL-13 levels.^8^ Interestingly, high CSF CXCL-13 levels have been reported to correlate with high intrathecal titers of anti-NMDAR antibodies, clinical severity at admission, relapses, worse treatment response, and greater long-term disability, highlighting its potential value as a prognostic and therapeutic biomarker.^24,25,27^ In addition, B-cell-activating factor (BAFF) and proliferation-inducing ligand (APRIL) are two other molecules from the TNF cytokine superfamily that play a role in B-cell survival, differentiation, and maturation, as well as in antibody synthesis.^30^ High serum and CSF levels of both molecules have been found in patients with NMOSD, Behçet disease, paraneoplastic opsoclonus-myoclonus (OM), myasthenia gravis (MG), systemic lupus erythematosus (SLE), Sjögren syndrome (SS), and rheumatoid arthritis (RA).^31–34^ Moreover, BAFF and APRIL levels were elevated in the acute phase of 40 patients with anti-NMDAR encephalitis and presented a positive correlation, thus reflecting a synergic role in neuroinflammation. Furthermore, high levels were associated with worse long-term outcomes.^35^ Conversely, other studies found no significant difference in BAFF and APRIL levels between anti-NMDAR patients and controls.^25,27,32^

**Table 2:** CSF cytokine profile of patients with anti-NMDAR encephalitis

Immune cell	Cytokine	Sample, n	Findings	References
**B-cell**	CXCL-13	271	Elevated in CSF during the acute phase	^24,25,27–29^
			Elevated in CSF during relapses	^24,27^
			Correlated with CSF titres of NMDAR antibodies	^24^
			Correlated with CSF pleocytosis	^25^
			Correlated with clinical severity	^27^
			Correlated with outcomes	^24,27^
	BAFF and APRIL	40	Elevated in CSF during the acute phase	^35^
			Associated with outcomes	^35^
**Th1**	IFN-γ	46	Elevated in CSF during the acute phase	^25,29^
			Persistently increased over months	^25^
	TNF-α	125	Elevated in CSF during the acute phase	^25,27,29,36–38^
			Persistently increased over months	^25^
			Only increased in CSF of paraneoplastic cases	^39^
	CXCL-10	30	Elevated in CSF during the acute phase	^25,27,29^
			Persistently increased over months	^25^
			Elevated in CSF during relapses	^27^
			Correlated with CSF pleocytosis	^25^
			Correlated with clinical severity	^27,29^
			Correlated with outcomes	^27^
	IL-2	24	Elevated in serum, which inversely correlated with CSF IL-6 and IL-17A	^26^
			Decreased significantly after treatment	^29^
**Th2**	CCL22	70	Elevated in CSF during the acute phase	^28,29^
			Correlated with clinical severity	^28,29^
**Th17**	IL-1β	109	Elevated in CSF during the acute phase	^28,40,41^
			Correlated NLRP3 levels	^40^
	IL-6	223	Elevated in CSF during the acute phase	^26–28,37,38,40,42,43^
			Correlated with clinical severity	^28,29,40,44^
			Correlated with and NLRP3 levels and HMGB1	^40,43^
	IL-7	52	Elevated in CSF during the acute phase	^25,29^
			Persistently increased over months	^25^
	IL-17A	189	Elevated in CSF during the acute phase	^25,26,28,40–43^
			Persistently increased over months	^25^
			Correlated with NLRP3 and HMGB1 levels	^40,43^
			Correlated with clinical severity	^40,42^
			Correlated with outcomes	^28^
			Elevated in CSF during relapses	^28^
**Treg**	IL-10	80	Elevated in CSF during the acute phase	^27,29,37,38^
			Only elevated in CSF of paraneoplastic cases	^39^
			Correlated with clinical severity	^45^

APRIL, proliferation-inducing ligand; BAFF, B cell-activating factor; CCL, C-C motif chemokine ligand; CSF, cerebrospinal fluid; CXCL, C-X-C motif chemokine; HMGB1, high-mobility group box 1; IFN, interferon; IL, interleukin; NLRP3, NOD-like receptor family, pyrin domain-containing 3; NMDAR, N-methyl-D-aspartate receptor; TNF, tumour necrosis factor; Th, T-helper cells; Treg, regulatory T cells.

Despite being mostly driven by humoral immunity, T cells may also have a role in anti-NMDAR encephalitis by orchestrating the ensuing inflammatory cascade and assisting B-cells in their maturation towards memory B-cells and long-lived plasma cells.^8,9,26,29^ Cellular immune responses recruit different T-cell subsets according to the nature of the antigen and the phase of the response.^11^ For instance, Th1-cells play a preferential role in the regulation of cytotoxic T cells and antibody-secreting cells (ASCs) through the production of different cytokines.^11^ Among them, TNF-α, which is broadly involved in the initiation and regulation of immune responses, was found at higher CSF concentrations in 125 patients with anti-NMDAR encephalitis compared to controls.^25,27,29,36–38^ Similarly, interferon-γ (IFN-γ), another pleiotropic cytokine produced by Th1-cells that stimulate ASC within the CNS by increasing their susceptibility to CXCL-10^9^, was elevated in the CSF of 46 anti-NMDAR patients.^25,29^ Moreover, IFN-γ was also persistently elevated in patients that developed anti-NMDAR encephalitis after herpetic encephalitis, suggesting that a long-lasting stimulation of B-cells could lead to the synthesis of autoantibodies.^46^ In addition, IFN-γ and TNF-α induce the expression of the receptor CXCR3 in immune cells as well as the secretion of its ligand CXCL-10, the main recruiter of Th1-cells into the CNS.^29^ Interestingly, CXCL-10 was elevated in the CSF of 30 patients with anti-NMDAR encephalitis,^25,27,29^ and remained elevated longer than CXCL-13, implying that after an initial B-cell response Th1-cell activation is needed to maintain the autoimmune reaction.^25^ Furthermore, the levels of CXCL-10 were associated with CSF pleocytosis, clinical severity, and relapses, reflecting its potential as prognostic biomarker.^25,27,29^ Strikingly, a single case report found that pleocytosis, CXCL-10, and CXCL-13 progressively increased in parallel with the patient’s clinical worsening until coma; furthermore, the increase in CXCL-10 levels preceded that of CXCL-13 and BAFF, suggesting a role of astrocytes in the initial phases.^9,25^ In addition, Th1-cells also produce IL-2, a pleiotropic cytokine that has a role in the proliferation of cytotoxic T cells, although it also induces anti-inflammatory feedback by stimulating the proliferation of Treg cells.^9^ IL-2 may limit uncontrolled inflammatory responses by interfering with IL-6-dependent signalling and inhibiting Th17-cell differentiation.^47^ Accordingly, high serum IL-2 levels negatively correlated with IL-6 and IL-17A CSF levels in a small cohort of patients with anti-NMDAR encephalitis.^26^ Surprisingly, although Th2-cells exert a major role in humoral immunity, only few chemokines involved in Th2-cell recruitment have been analysed in anti-NMDAR encephalitis.^28,45^ Among them, C-C motif chemokine ligand (CCL) 22 was elevated in 70 patients,^28,29^ and its levels positively correlated with clinical severity.^29^ Moreover, CCL22 plays a role in the recruitment of Th17-cells, reflecting its pleiotropic properties and supporting a synergic role of both types of T-cell responses in anti-NMDAR encephalitis.^28^

An increased TH17/Treg ratio is considered a hallmark of autoimmunity and is associated with disease activity in psoriasis, inflammatory bowel disease, RA, and multiple sclerosis.^4^ Interestingly, high CSF Th17-cells levels have also been found in 60 patients with anti-NMDAR encephalitis.^28^ The balance between Th17 and Treg-cells is tightly regulated by the surrounding cytokine microenvironment: in the presence of TGF-β and IL-2, naïve CD4+ T cells polarize into Treg-cells, which mainly produce anti-inflammatory cytokines such as IL-10, while TGF-β and IL-6 drive the differentiation into Th17-cells that mainly produce IL-1β, 6, 7, 8, and 17.^4,7^ The activation of nucleotide-binding oligomerization domain-like receptor family pyrin domain-containing 3 (NLRP3) inflammasome induces the migration of Th1 and Th17 cells into the CNS *via* IL-1β production, which was found elevated in the CSF of 109 patients with anti-NMDAR encephalitis.^28,40,41^ As it has been mentioned before, IL-6 is a pleiotropic cytokine that regulates Th17/Treg balance, stimulates B-cell differentiation and antibody production, and regulates the survival of plasmablasts implicated in CNS disorders.^48^ High intrathecal IL-6 levels have been found in 223 patients with anti-NMDAR encephalitis, which were associated with a worse clinical status.^26–29,37,38,40,42,44^ Additionally, IL-7, a critical survival factor of regular and autoreactive T cells, was found to be increased in 52 patients with anti-NMDAR encephalitis.^25,29^ Furthermore, IL-17A, a cytokine that stimulates the expression of IL-6 and facilitates leukocyte trafficking across the blood-brain barrier (BBB),^43^ was found to be elevated in 189 anti-NMDAR patients and associated with clinical severity, relapses, and outcomes.^25,26,28,40–42,44^ Therefore, novel immunotherapies aimed to restore the Th17/Treg-cell balance could be promising in future therapeutic strategies for anti-NMDAR encephalitis.

However, some of these cytokine deviations are not exclusive of anti-NMDAR encephalitis, and may partially overlap with other neuroimmune and neuroinfectious disorders, such us TNF-α, IL-6, IL-10, CXCL-13, CXCL-10, and IL-1. These findings suggest shared immunological pathways involving humoral and cellular immunity, and might offer novel treatment strategies for both immune and infectious disorders.^27^

#### Anti-leucine-rich glioma-inactivated 1 encephalitis

Anti-leucine-rich glioma-inactivated 1 (LGI1) encephalitis is characterized by the presence of antibodies against the glycoprotein LGI1, predominantly of the IgG4 subclass although IgG1 is also frequent in both serum and CSF.^49^ LGI1 is a secreted protein that interacts with presynaptic ‘A disintegrin and metalloproteinase domain-containing protein’ (ADAM) 23 as well as postsynaptic ADAM22, regulating the function of presynaptic Kv1.1 potassium channels and postsynaptic α-amino-3-hydroxy-5-methyl-4-isoxazolepropionic acid receptor (AMPAR).^50,51^ Interestingly, nearly 20% of patients have LGI1 antibodies detectable only in the serum, and 50% have no inflammatory CSF abnormalities, likely reflecting a different immune mechanism compared to other AE driven by neural cell-surface antibodies such as anti-NMDAR encephalitis.^49^ These findings are in agreement with the scarce lymphocytic infiltrates and immunoglobulin deposition described in histopathological studies. However, mild B- and T-cell infiltrates and heavy IgG and complement deposits may be found, leading to focal areas of neuronal loss that may explain the frequent residual cognitive deficits^21,52–54^.

In concordance with the scarce inflammatory reaction distinctive of anti-LGI1 encephalitis, only a few studies found significant differences in intrathecal cytokine levels compared to controls.^55,56^ High CSF levels of CXCL-13 were observed despite an unremarkable CSF analysis, highlighting that a humoral CNS immune response may be present even in the absence of an evident inflammatory CSF. However, serum levels were generally higher, indicating a peripheral synthesis and a subsequent leakage into the CNS.^55,57^ In addition, Th17-cell-related cytokines such as IL-17A were increased in patients with anti-LGI1 encephalitis, and associated with disease severity and risk of ICU admission at onset,^56^ suggesting that cellular immunity may also play a pathogenic role, likely assisting B-cells in antibody production.^55^ However, other studies failed to find significant differences in CSF cytokine levels compared to controls.^26^

#### Other encephalitides

The cytokine profile of AE associated with antibodies against Hu, Yo, glutamic acid decarboxylase (GAD), γ-Aminobutyric acid-B (GABA_B_) receptor, GABA_A_ receptor, contactin-associated protein-like 2 (CASPR2), immunoglobulin-like cell adhesion molecule 5 (IgLON5), and adenylate kinase 5 (AK5) have also been explored in several studies^32,55,56,58,59^. However, no firm conclusions can be drawn due to the small size of the samples and the different nature of these diseases. Moreover, the heterogeneity of involved cytokines and pathways hamper the understanding of actionable pathogenic mechanism.^32,55,56,58,59^ Nevertheless, it is noteworthy that patients with anti-Yo and anti-Hu paraneoplastic cerebellar degeneration had enrichment of CXCR3+ T cells and CXCL-10 in the CSF, supporting a major role of cellular immunity in the pathogenesis of these disorders.^58^

### A general approach to targeted immunotherapies

The current therapeutic approach of AE is based on retrospective studies suggesting a benefit of immunotherapy, but no prospective clinical trials have been conducted yet in AE to support this practice.^60^ Consequently, most immunotherapies in AE are given empirically and in a non-specific manner, independently of the suspected underlying pathogenic mechanisms. For instance, after the failure of the so-labelled first-line therapies, it is frequent to combine ASC-depleting agents, such as rituximab, with a wide-spectrum drug such as cyclophosphamide, in both antibody and T-cell mediated AE.^61^ However, this strategy likely increases the risk of side effects and may even affect the anti-tumour response in paraneoplastic cases. Hence, novel therapeutic regimens tailored to modulate the specific underlying immune response are required to improve outcomes and prevent undesired events in patients with AE.

**Figure 1:**
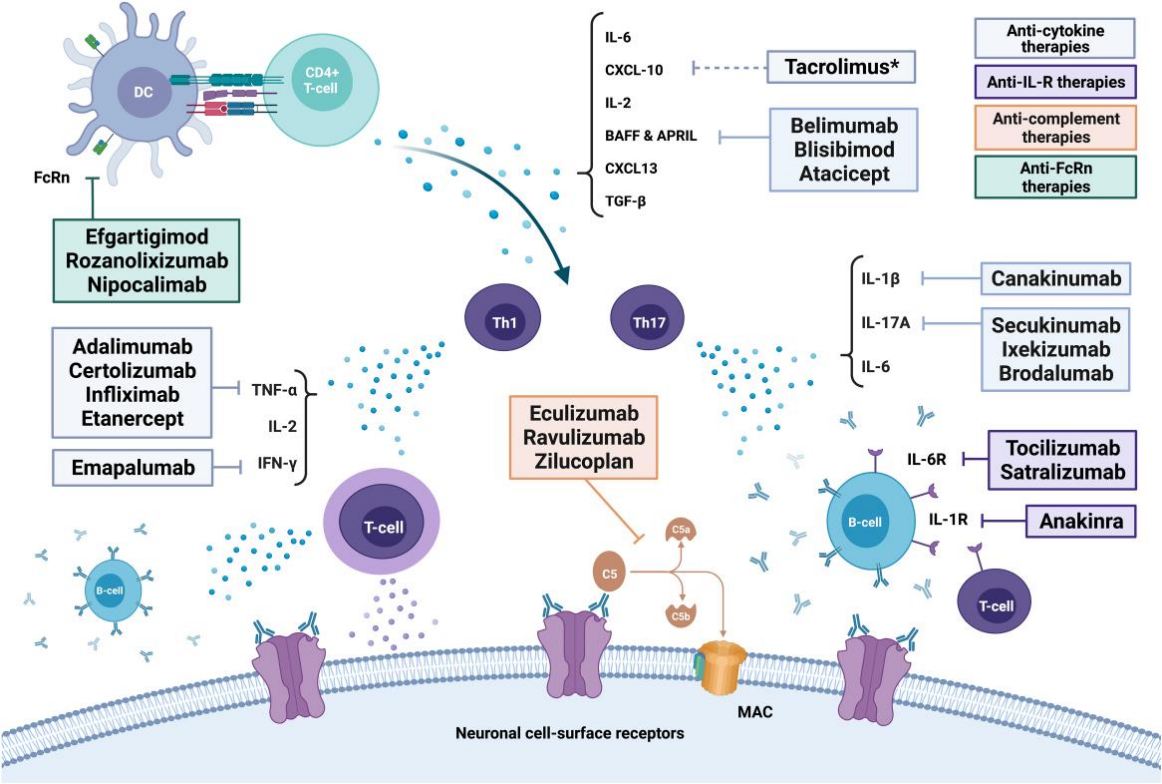
**Targeted immunotherapies against soluble biomarkers.** *Tacrolimus is not selectively directed against CXCL-10 but it has been reported to decrease intrathecal CXCL-10 levels. APRIL, proliferation-inducing ligand; BAFF, B-cell-activating factor; CXCL, C-X-C motif chemokine; DC, dendritic cells; FcRn, neonatal Fc receptor; IFN, interferon; IgG, immunoglobulin G; IL, interleukin; IL-R, interleukin receptor; MAC, membrane attack complex; NMDAR, N-methyl-D-aspartate receptor; TGF, transforming growth factor; Th, T-helper cells; TNF, tumour necrosis factor.

**Figure 2:**
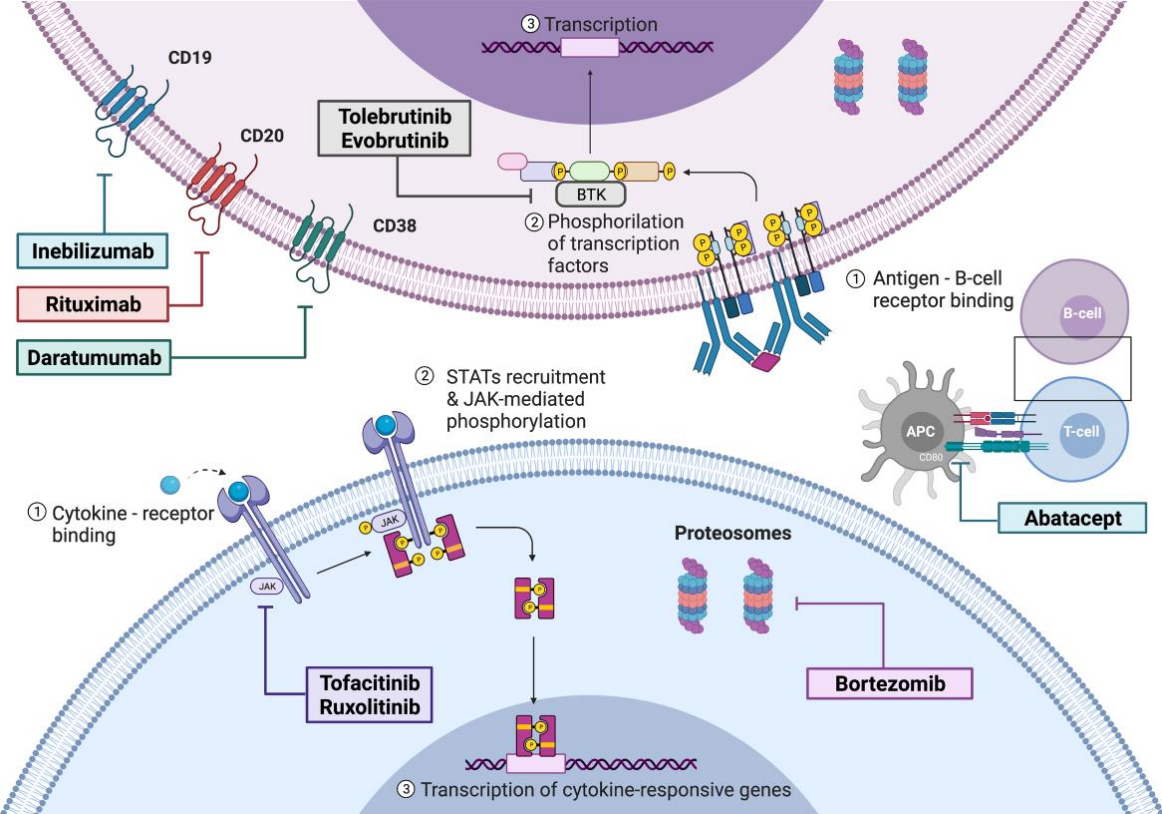
**Targeted immunotherapies against membrane or intracellular proteins.** APC, antigen-presenting cell; BTK, Bruton’s tyrosine kinases; JAK, Janus kinases; STAT, signal transducers and activators of transcription.

Countless novel immunotherapies have recently been developed to block specific immune pathways at different points of the signalling cascade driving neuroinflammation. A first approach is to deplete specific immune cells subpopulations such as B-cells, plasma cells, or cytotoxic T cells, by targeting their specific cluster of differentiation (CD) with monoclonal antibodies (mAbs), or interfering with their metabolism and inducing apoptosis. Secondly, because excessive recruitment of leukocytes and lymphocytes to affected tissues and organs is a key feature of autoimmune disorders, specific inhibition of this process would be an ideal anti-inflammatory strategy. Therefore, circulating signalling molecules such as cytokines and chemokines produced by immune cells may also be targeted by soluble receptors, neutralizing proteins or mAbs in order to prevent the interaction with their CKR (Fig. 1). Additionally, recombinant fusion proteins and mAbs can be directed against these receptors to prevent their activation and subsequent intracellular signalling cascades or even antagonize their function. These intracellular pathways comprise several cytoplasmic proteins that can also be selectively inhibited to prevent the transcription of selected genes (Fig. 2). Lastly, soluble immune effectors, including antibodies and complement proteins, could also be removed or blocked by mAbs and fusion proteins.

### Potential targeted immunotherapies in autoimmune encephalitides

In this section, we comprehensively review the potential therapeutic options to modulate the immune mechanisms driving AE from a theoretical point of view according to the results presented in the section entitled ‘The role of cytokine networks in AE’. We then summarize the current knowledge regarding their use in other systemic and neurological immune disorders, and, if available, experience in selected patients with AE. All these treatments are further classified according to their predominant effect on the humoral or cellular immunity, the nature of the targeted molecule, and the immune pathways in which they are involved. Supplementary Table 1 presents the main immunotherapies considered potentially relevant in the near future for the management of AE.

### Humoral immunomodulators

Several agents have recently been used in antibody-mediated disorders to modulate selectively the humoral immune system. Herein, we describe these therapeutic alternatives according to their mechanisms, which include acting directly on ASC, blocking soluble mediators relevant for B-cell proliferation, removing the final effectors of the humoral response, and modulating intracellular signalling inhibitors such as Bruton’s kinase (BTK) inhibitors.

#### Antibody-secreting cells depletion therapies

Autoimmune CNS disorders mediated by antibodies have successfully been treated with B-cell depleting therapies such as rituximab in large retrospective.^62,63^Another anti-CD20 agent, ocrelizumab, has also been suggested to be effective in a prospective clinical trial for AE, although it was prematurely closed due to poor recruitment^64^, which highlights the need of international collaborative clinical trials to achieve enough sample sizes to assess alternative immunotherapies in AE. However, it has been suggested that refractory cases are driven by CD19 + CD20- long-lived plasma cells (LLPC), explaining why some cases refractory to rituximab respond to plasma cell depletion therapies such as anti-CD19 or anti-CD38 agents.^65–67^ Accordingly, intrathecal CD20 + memory B-cells, CD38+ ASC, and CD19 + CD138 + plasma cells have been found in patients with anti-NMDAR, anti-GABAbR, and anti-LGI1 encephalitis.^20,68,69^ Therefore, novel anti-CD19 therapies have been proposed to treat antibody-mediated disorders such as NMOSD, for which inebilizumab, a mAb against CD19, has recently been approved^70^ and is soon going to be assessed in a trial for anti-NMDAR encephalitis (NCT04372615), while chimeric antigen receptor-T (CAR-T) cells targeting CD19+ B-cells are currently being assessed in a clinical trial for NMOSD (NCT03605238) and MG (NCT04146051). However, anti-CD19 CAR-T cells have been reported to enhance disease progression in a mouse model of multiple sclerosis.^71^ These therapies could lead to a broad depletion of late B-cell stages including ASC, plasmablasts, and early plasma cells; however, terminally differentiated plasma cells may not be depleted as they can lose the expression of CD19 and CD20.^72^ In order to remove these terminally differentiated plasma cells, other therapies used for multiple myeloma have been proposed; for instance, daratumumab, which targets CD38, has been reported to be effective in small case series of anti-NMDAR and anti-CASPR2 encephalitis.^73,74^ This selective depletion of ASC may lead to higher effectiveness and safety compared to conventional anti-CD20 therapies. However, there is limited evidence of the impact of ASC-depleting therapies on intrathecal or parenchymal ASC, especially relevant in AE with B-cell CNS infiltration and intrathecal antibody synthesis.

#### Cytokine-targeted immunotherapies

Among the different types of drugs directed against cytokines, anti-IL therapies are the most relevant since they have revolutionized the management of several immune and infectious diseases such as RA or psoriasis, and even more recently COVID-19.^75^ IL-6 is a tempting target for antibody-mediated disorders as the blockage of its receptor restricts B-cell antibody production. Tocilizumab is an anti-IL-6R mAb already used for RA and other juvenile arthritis, giant cell arteritis, and CAR-T cell-induced cytokine release syndrome.^76^ Regarding AE, tocilizumab has been reported to be effective in nearly 100 patients unresponsive to rituximab, showing higher effectiveness compared to extended administration of rituximab and a surprisingly fast response (within the first weeks).^77–79^ In addition, the administration within the first month of corticosteroids, intravenous immunoglobulins, rituximab, and tocilizumab, along with teratoma removal if present (T-SIRT protocol) was associated with a better outcome at 1-year follow-up in anti-NMDAR encephalitis.^80^ However, adverse events such as leukopaenia and pneumonia were commonly observed with this strategy.^80^ Although there is limited evidence to broadly recommend tocilizumab in AE, recent expert consensus recommendations are considering its use in severe, refractory adult and paediatric cases.^61,81^ Another mAb against the IL-6R is satralizumab, which has recently been approved for NMOSD due to its enhanced antigen-specificity and a longer plasma half-life than tocilizumab.^82^

A different approach to modulate humoral immunity is the blockade of BAFF and APRIL pathways with several agents that have mainly been used in SLE. Belimumab is an anti-BAFF mAb that has been reported to be effective and safe in patients with SLE.^83^ Conversely, it was not effective for MG in a recent clinical trial,^84^ although patients with antibodies against muscle-specific tyrosine kinase (MuSK) were not included, and they likely represent the subgroup more prompt to respond to anti-B-cell therapies.^85^ Additionally, blisibimod is a molecule with features of both peptides and antibodies that exerts a selective BAFF antagonism that has been reported to be effective in SLE.^86^ Although no therapies have been developed to exclusively target the APRIL pathway, atacicept is a human recombinant fusion protein performing a dual blockade of BAFF and APRIL that increased relapses in patients with multiple sclerosis, hence its benefit in other immune CNS disorders remains unclear.^87^

In addition, novel mAbs against the major chemokine involved in B-cell recruitment and Th17-cell regulation, CXCL-13, have been reported to have good tolerance and efficacy in murine models of relapsing-remitting experimental autoimmune encephalomyelitis, RA, and lupus nephritis, suggesting that they could be useful in the treatment of their homologous human disorders.^88,89^ In addition, combining BAFF and CXCL-13 inhibitors may constitute another synergic strategy for blocking B-cell pathways, which was found to have higher efficacy in a murine model of SS than targeting BAFF alone.^90^ Thus, the combination of different immunotherapies that synergically block specific immune pathways in a tailored manner could be a promising strategy in the management of AE.

#### Antibody-targeted therapies

Humoral immunity can also be modulated directly by interfering with its main effectors. Interestingly, the neonatal Fc receptor (FcRn) system allows the recycling of serum IgGs, avoiding lysosomal degradation, by transporting them back to circulation and prolonging their survival.^91^ Thus, reversing this process with FcRn antagonists decreases IgG levels up to 85% from baseline without affecting other immune cells, therefore preventing severe and complete immunosuppression.^92^ Efgartigimod is an engineered human IgG1 antibody Fc-fragment designed to block the FcRn that has been reported to cause a rapid and long-lasting improvement in 75% of patients with MG.^93^ Novel mAbs have been designed to recognize FcRn, such as rozanolixizumab and nipocalimab, leading to a rapid depletion of 75–90% of IgG levels.^94^ These therapies are showing promising results in MG (NCT0397142, NCT04951622),^95^ which led to the design of a new clinical trial with rozanolixizumab for anti-LGI1 encephalitis (NCT04875975). However, the application of these therapies in AE could be controversial as FcRn plays a different role in the CNS, where it removes intrathecal IgG by transporting them to the circulation through reverse transcytosis across the BBB.^96^ Further research to selectively block peripheral FcRn or stimulate central FcRn could be promising in the future management of AE and other CNS antibody-mediated disorders.

#### Complement-targeted therapies

The complement system is an essential element of both innate and adaptive immune responses that amplifies the function of phagocytes and antibodies. Anti-complement therapies are effective in systemic and neurological autoimmune disorders, suggesting its potential benefit in AE with evidence of complement activation.^85,97^ Eculizumab, an anti-C5 mAb that inhibits the C5 cleavage into C5a and C5b preventing complement activation, has recently been approved for refractory anti-acetylcholine receptor MG and anti-aquaporine 4 NMOSD.^98,99^ In addition, ravulizumab, a second-generation anti-C5 mAb with a longer plasma half-life, is also currently undergoing a clinical trial for MG (NCT03920293). Another therapy to inhibit complement-mediated tissue damage currently being evaluated in MG is zilucoplan, a synthetic macrocyclic peptide that binds C5 with sub-nanomolar affinity inhibiting the cleavage into C5a and C5b (NCT04115293).^94^ Although anti-complement immunotherapies are promising for AE with complement activation such as anti-AK5 AE,^100^ their efficacy in IgG4-mediated disorders is unclear, as this IgG subclass cannot theoretically activate complement due to an inefficient binding to C1q.^97^ However, this assumption should be carefully considered since complement deposition has been described in histopathological studies of AE traditionally thought to be driven by IgG4, such as anti-LGI1 and anti-CASPR2 encephalitis,^21,101^ which moreover commonly present other IgG subclasses in serum and CSF such as IgG1.^49,102^

#### Therapies targeting B-cell intracellular signalling molecules

Alternative agents proposed for the management of autoimmune CNS disorders are BTK inhibitors, mainly due to their ability to cross the BBB.^103^ BTK are cytoplasmic tyrosine kinases that play a central role in B-cell development and production of cytokines and antibodies by regulating the interaction between specific cell-surface receptors and their downstream signalling pathways.^104^ BTK is present in most haematopoietic cells except for T-, natural killer-, and plasma cells. Therefore, they have primarily been used in B-cell malignancies, although evobrutinib and tolebrutinib have been reported to have good efficacy and tolerability in multiple sclerosis.^105,106^ Since BTK is not present in plasma cells, BTK inhibitors could be useful in the early phases of AE that are predominantly driven by humoral responses, but not in late stages when plasma cells are responsible for antibody production.

### Cellular immunomodulators

As reviewed in the section entitled ‘The role of cytokine networks in AE’, the cellular immunity seems to play a key role not only in AE associated with antibodies against intracellular antigens, but also in AE driven by neural cell-surface antibodies by assisting humoral immune cells in antibody production. A broad variety of agents that modulate different points of the cellular immune cascade are summarized in the present section, including T-cell depleting therapies, cytokine and CKR blockers, and intracellular signalling inhibitors as JAK inhibitors.

#### T-cell depleting therapies

A decrease in T-cell populations can be achieved through a wide variety of therapies. However, most of these therapies are not selective for T cells and also affect B-cell populations. Cyclophosphamide is an alkylating antineoplastic agent used in several autoimmune disorders due to its ability to slow T-cell proliferation,^107^ and its use in AE is supported by solid evidence from retrospective studies.^62^ Bortezomib is a reversible ubiquitin-proteasome inhibitor used in lymphomas and multiple myeloma, and which induces apoptosis of activated T cells and antibody-secreting plasma cells.^108,109^ Small series have shown its efficacy in anti-NMDAR encephalitis after the failure of more widely used treatments,^110–112^ yet some physicians select it instead of cyclophosphamide in young patients.^113^ However, there is currently no solid evidence supporting its wide application but a clinical trial in AE will soon provide additional data (NCT03993262). Another option reported to be effective in small series of anti-NMDAR encephalitis is intrathecal methotrexate, a folate antagonist widely used in haematological malignancies that inhibits the enzyme dihydrofolate-reductase. Its intrathecal administration could be an asset in AE with intrathecal synthesis of antibodies, but further studies should assess its safety and efficacy.^25,114–116^ Another T- and B-cell depleting therapy proposed for AE owing to the previous experience in multiple sclerosis is alemtuzumab, a mAb against CD52.^117^ However, its use should be carefully considered as its main safety concern is the development of secondary autoimmune disorders including anti-GABA_A_ receptor and anti-glutamate receptor 3 (GluR3) AE.^118,119^

#### Cytokine-targeted immunotherapies

Anti-TNF-α mAbs such as adalimumab, certolizumab, and infliximab, as well as etanercept, a recombinant fusion protein that blocks circulating TNF-α, are used in several autoimmune and inflammatory disorders including RA, Crohn’s disease, ankylosing spondylitis, and neurosarcoidosis.^120^ Despite being effective and overall safe for these indications, they have been associated with central and peripheral demyelinating diseases, lupus-like syndromes, and vasculitis.^121^ Hence, although they could be effective in AE, further studies should clarify the nature of these adverse events before their application in immune CNS disorders. Likewise, IFN-γ can also be targeted by emapalumab, a mAb successfully used in patients with primary hemophagocytic lymphohistiocytosis.^122^

Since a Th17/Treg-cell imbalance in favour of the former has been related to the development of autoimmunity, the blockade of cytokines that drive Th17-cell proliferation could be a future therapeutic approach in AE.^123^ IL-1β can be targeted by canakinumab, a mAb used to treat cryopyrin-associated periodic syndromes and gout.^124^ Moreover, IL-1R can be antagonized with anakinra, a recombinant protein that has been reported to attenuate seizures in an animal model of anti-NMDAR encephalitis,^125^ and in patients with new-onset refractory status epilepticus (NORSE) as well as those with febrile infection-related epilepsy syndrome (FIRES),^126,127^ two poorly understood entities that might have some pathogenic similarities with AE. Interestingly, IL-1 blockage is considered to promote anti-tumour immunity and synergize with immunotherapy, which could be particularly useful for paraneoplastic cases.^128,129^ Additionally, anti-IL-6 therapies are also feasible candidates not only to modulate cellular immunity, but also to treat paraneoplastic cases as they seem to enhance anti-tumour immunity.^128,130^

Similarly, IL-17 can be targeted with mAbs such as secukinumab, a therapy broadly used in psoriasis and ankylosing spondylitis that seems to be also effective in multiple sclerosis,^131^ suggesting its potential benefit in AE. In addition, novel anti-IL17A therapies such as ixekizumab and brodalumab could also be proposed to antagonize this pathway, although the use of anti-IL17A therapies in paraneoplastic cases should be carefully considered until further studies clarifies its impact on anti-tumour immune response.^132^

Moreover, the administration of cytokines with anti-inflammatory properties such as IL-2 or IL-10 could also be used to ensure the balance between Th17- and Treg-cells.^133^ Indeed, the administration of low doses of IL-2 to selectively stimulate Treg cells has been reported to be safe and effective for refractory AE,^134^ as Treg cells present a lower activation threshold than T cells.^135^ Similarly, daclizumab, which is a mAb acting as an agonist of the IL-2R, was reported to normalize the proportions of T and B cells in the CSF of patients with multiple sclerosis.^136^

In addition, several immunotherapies directed against other cytokines such as IL-12/IL-23 could also be applied in AE. However, we consider these treatments out of the scope of this review since there is currently little evidence regarding the role of these cytokines in AE or in other neuroinflammatory contexts.

#### Therapies targeting intracellular signalling molecules

JAKs are multidomain non-receptor tyrosine kinases that govern intracellular cytokine signals relevant for B- and T-cell proliferation and maturation by regulating transcription and gene expression.^137^ Since all CKR transmit their signals through JAK1, JAK2, JAK3, or TYK2, their selective blockade with JAK inhibitors allows the simultaneous modulation of multiple cytokine signalling pathways. Moreover, due to their ability to cross the BBB, they offer an interesting therapeutic option from a theoretical point of view, although the evidence regarding their efficacy in neuroimmune disorders is scarce. Tofacitinib is a JAK 1–3 inhibitor used in RA, psoriasis, and ulcerative colitis that seemed to be effective in selected cases of refractory AE,^138^ whereas ruxolitinib, a dual JAK1 and JAK2 inhibitor approved for myelofibrosis, was effective in single case reports of anti-MuSK MG, NMOSD and immune checkpoint inhibitor-induced myelitis.^139^ Therefore, this multi-cytokine blockade seems promising for AE, although its administration in patients with active cancer is currently not recommended due to concerns about its impact on anti-tumour immune response and effectiveness of immunotherapy. JAK inhibitors have already been approved for multiple rheumatological conditions and they are currently being evaluated in several clinical trials for solid non-haematological malignancies.^140–145^.

Furthermore, the blockade of signalling molecules responsible for T-cell recruitment into the CNS could be applied in the initial phases of AE to prevent the full development of the immune response. Tacrolimus, a calcineurin inhibitor used in several autoimmune disorders, has been reported to selectively deplete CSF CXCL-10 in rapidly progressive cerebellar syndrome associated with Yo and Hu antibodies.^58^

### Other targeted immunotherapies

The recruitment and migration of immune cells across the BBB is a crucial process orchestrated by multiple chemokines and other molecules involved in neuroinflammation. Thus, agents preventing the entry of T cells into the CNS could be useful in AE. Natalizumab, a mAb against the subunit α4 of adhesion integrins, has been reported to reduce intrathecal and peripheral levels of several cytokines in patients with multiple sclerosis, and could therefore potentially be effective in AE, particularly in paraneoplastic cases in which the peripheral anti-tumour response should not be compromised.^146^ Nevertheless, hampering the CNS immune surveillance could increase the risk of CNS metastasis. Recently, a Phase II trial in Hu-associated paraneoplastic syndromes found partial improvement with natalizumab, although most patients had very severe subacute sensory neuropathies.^147^

Another promising strategy is the positive modulation of immune checkpoints to increase peripheral self-tolerance and decrease self-reactive immune responses. Abatacept is a fusion protein composed by cytotoxic T-lymphocyte antigen 4 (CTLA4) and the Fc region of IgG1 that binds to CD80 or CD86, thus suppressing the second T-cell activation signal and downregulating cellular immunity. In addition, abatacept may also modulate B-cell activity by internalizing CD80/CD86 and decreasing plasmablasts and IgG levels.^148^ Hence, it seems likely that its applicability in AE and neurological complications of immune checkpoint inhibitors could be effective, although previous reports in immune checkpoint inhibitor-induced myocarditis found opposing results.^149^

### Conclusions and future perspectives

Recent improvements in cytokine identification and quantification provide new insights into the immune mechanism underlying neuroinflammation. In this review, we summarized several studies that explored their dynamics and suitability as prognostic and therapeutic biomarkers in AE, chiefly anti-NMDAR and anti-LGI1 encephalitis, reflecting the synergic pathogenic role of humoral and cellular immunity. However, the interpretation and comparison of the aforementioned studies is challenging due to several methodological limitations that should be considered in the design of future cytokine studies.

First, since AE are rare conditions, most studies included a small sample of patients and therefore solid conclusions cannot be reached. However, the incidence of AE has significantly increased in the last decade probably due to improvements in their diagnosis.^150,151^ Second, the CSF/serum cytokine gradient and BBB dysfunction were rarely reported despite being crucial to determine intrathecal production. Hence, future studies should study cytokines in paired CSF/serum samples at similar time-points, as the concentration and location of cytokines may change during the course of the disease and after the administration of immunomodulators. Otherwise, the results obtained may lead to misinterpretations that might explain the high heterogeneity observed so far. Furthermore, the use of cytokines as biomarkers is challenging due to their complex nature, and their interpretation requires the understanding of their pleiotropic activities, interindividual variability, dynamics in relation to the course and severity of the disease, and their interactions with cytokine-binding proteins and soluble receptors that may underestimate their quantification.^152^ Thus, all these confounding factors should be considered when designing new cytokine studies and tailoring immunotherapeutic strategies for AE.

Currently, AE are treated empirically and independently of the underlying pathogenic mechanisms, mainly due to the absence of clinical trials on these rare disorders. However, the increasing knowledge on the cytokine dynamics summarized in this review offers a promising opportunity to treat patients in a personalized manner that could change the present paradigm of AE management. In the era of ‘biomarker-guided therapy’, cytokines might be useful in the diagnosis of neuroimmune disorders and to identify subsets of patients that could potentially benefit from novel therapeutic strategies, thus improving outcomes and minimizing side effects.^153,154^ However, these new therapies should be administered as early as possible in the disease course to avoid misinterpretation, since their targets are dynamic and activate downstream signalling cascades that may underestimate their efficacy if administered in an advanced stage of the disease. Additionally, the blockade of a sole pathway may be insufficient to control the entire immune reaction, especially if not applied before the full response has been developed. Indeed, the simultaneous blockade of cytokines and their CKR, or the concomitant blockade of inflammatory pathways and activation of anti-inflammatory processes have been proposed to be more effective.^5^ Similarly, the interruption of intracellular signalling pathways relevant for multiple cytokines with JAK or BTK inhibitors, instead of a single pathway inhibition, could be more effective. Hence, future therapeutic strategies for AE should comprise combinations of immunotherapies to regulate various key points of the immune system considering the clinical characteristics and comorbidities of the patient, such as the type of neural antibodies associated, cancer, and cytokine profile, in order to tailor personalized synergic therapies directed against B- and/or T cells, cytokines or CKRs, antibodies, complement, or specific signalling molecules.

Future studies on the cytokine profile of AE should comprise international, collaborative, prospective designs to avoid current limitations related to small samples and cytokine dynamics during the course of the disease. Concurrently, prospective clinical trials assessing the safety and efficacy of novel targeted immunotherapies are required to reach stronger evidence and improve outcomes in patients with AE.

## Supplementary Material

fcac196_Supplementary_Data

## Abbreviations

ADAM =: A disintegrin and metalloproteinase domain-containing protein
AE =: autoimmune encephalitides
AK5 =: adenylate kinase 5
AMPAR =: α-amino-3-hydroxy-5-methyl-4-isoxazolepropionic acid receptor
APRIL =: proliferation-inducing ligand
ASC =: antibody-secreting cell
BAFF =: B cell-activating factor
BBB =: blood-brain barrier
BTK =: Bruton’s tyrosine kinase
CAR-T =: Chimeric Antigenic Receptor-T
CASPR2 =: contactin-associated protein-like 2
CCL =: C-C motif chemokine ligand
CKR =: cytokine receptor
CXCL =: C-X-C motif chemokine
FcRn =: neonatal Fc receptor
GAD =: glutamic acid decarboxylase
HMGB1 =: high–mobility group box 1
IFN =: interferon
IgLON5 =: immunoglobulin-like cell adhesion molecule 5
IL =: interleukin
JAK =: Janus kinase
LGI1 =: leucine-rich glioma-inactivated 1
mAb =: monoclonal antibody
MG =: myasthenia gravis
MUSK =: muscle-specific tyrosine kinase
NMDAR =: N-methyl-D-aspartate receptor
NMOSD =: neuromyelitis optica spectrum disorder
NLRP3 =: NOD-like receptor family, pyrin domain-containing 3
OM =: opsoclonus-myoclonus
RA =: rheumatoid arthritis
SLE =: systemic lupus erythematosus
SS =: Sjögren syndrome
STAT =: signal transducers and activators of transcription
TGF =: transforming growth factor
TNF =: tumour necrosis factor
Th =: T-helper cells
Treg =: regulatory T cells
