# Supplementary material for: Cytokine dynamics and targeted immunotherapies in autoimmune encephalitis
Source: Brain Commun. 2022 Aug 20;4(4):fcac196. doi: 10.1093/braincomms/fcac196 (PMC9392471; doi:10.1093/braincomms/fcac196)
Supplement: fcac196_Supplementary_Data [file fcac196_supplementary_data.docx]

Supplementary table

Supplementary table 1: Targeted-immunotherapies proposed for AE.

| **T- or B-cell depleting therapies** | | | | | |
| --- | --- | --- | --- | --- | --- |
| **Drug** | **Mechanism** | **Dosage** | **Pretreatment test** | **Monitoring** | **Side effects** |
| **Rituximab**^30^ | Anti-CD20 mAb | 375 mg/m²/week IV for 4 weeks  1 g twice within 15 days  Premedication: antipyretics and antihistamines | CBC, liver function, serologies (hepatitis, HIV, TB, VZV), serum Igs levels | CBC with differential, liver function, infection assessment | Infusion reactions, upper respiratory tract infections, PML, RPLS, severe heart failure, hepatitis B and herpes reactivation, malignancy risk, immunoglobulin deficits |
| **Inebilizumab**^160^ | Anti-CD19 mAb | 300 mg IV on days 0 and 15, then every 6 months  Premedication: steroids, antipyretics and antihistamines | CBC, liver function, serologies (hepatitis, HIV, TB, VZV), serum Igs levels | Infection assessment | Urinary tract infection, arthralgia, infusion-related reaction, nasopharyngitis, headache, neutropenia |
| **Bortezomib**^161^ | Proteasome inhibitor, induces apoptosis of activated T-cells and plasma cells | 1.3 mg/m² IV on days 1-4-8-11 of a 21- day cycle, with concomitant dexamethasone administration. Maintained every 3 weeks or monthly  Prophylaxis with acyclovir and cotrimoxazole | CBC, liver function, serologies (hepatitis, HIV, TB, VZV) | CBC with differential, liver function, peripheral nerve clinical assessment, periodic pulmonary function testing | Digestive disturbances, peripheral neuropathy, cytopenias, hypotension, hepatotoxicity, hepatitis B and herpes reactivation, PML, pericardial disorders, interstitial infiltrative lung disease |
| **Intrathecal methotrexate**^162^ | Folate antagonist | 10 mg methotrexate + 10mg dexamethasone weekly for 1 month | CBC, liver and renal function, serologies (hepatitis, HIV, TB) | CBC with differential, liver and renal function, pulmonary function testing, infection assessment | Acute chemical arachnoiditis, chronic leukoencephalopathy, subacute myelopathy |
| **Daratumumab**^117^ | Anti-CD38 mAb | 16 mg/kg/weekly IV for 8 weeks, then biweekly  Premedication: steroids, antipyretics and antihistamines | CBC, serologies (hepatitis, HIV, TB)  Interference with cross-matching and red blood cell antibody screening. Test patients prior to starting treatment. | CBC with differential, infection assessment | Infusion reactions, cytopenia, diarrhea, constipation, vomiting, muscle spasms, arthralgia, pyrexia, dizziness, insomnia, cough, dyspnea, peripheral edema, peripheral sensory neuropathy and upper respiratory tract infection |
| **Cytokine-targeted therapies** | | | | | |
| **Drug** | **Mechanism** | **Dosage** | **Pretreatment test** | **Monitoring** | **Side effects** |
| **Canakinumab**^163^ | Anti-IL1β | 150-300 mg SC every 4-8 weeks | CBC, liver function, serologies (hepatitis, HIV, TB) | CBC, liver function, infection assessment | Upper respiratory tract infections, diarrhea, nausea, headache, gastroenteritis, weight gain, vertigo |
| **Anakinra**^164^ | IL1R antagonist | 1-10 mg/kg/day SC | CBC, liver function, serologies (hepatitis, HIV, TB) | CBC with differential, infection assessment | Injection-site reaction, upper respiratory tract infection, headache, nausea, diarrhea, arthralgia, flu-like symptoms, abdominal pain |
| **IL-2**^165^ | IL-2R ligand | 1.5 million IU/day SC for 5 days, followed by three 5-day courses of 3 million IU/day at weeks 3, 6, and 9 | CBC, liver function, serologies (hepatitis, HIV, TB) | CBC with differential | Injection-site reactions, flu-like symptoms, nausea, diarrhea, neutropenia, and eosinophilia, |
| **Daclizumab**^138^ | IL-2R agonist | 150 mg SC monthly | CBC, liver function | CBC with differential, liver function | Severe liver injury (autoimmune hepatitis, liver failure), skin reactions, lymphadenopathy, non-infectious colitis, depression, upper respiratory tract infections. |
| **Tocilizumab**^166^ | Anti-IL-6R mAb | 8 mg/kg (12mg/kg for patients <30kg) IV, then monthly IV doses, or weekly SC 162mg | CBC, liver function, serologies (hepatitis, HIV, TB) | CBC with differential, liver function, lipid profile, infection assessment | Upper respiratory tract infections, headache, hypertension, hepatotoxicity, injection-site reactions, gastro-intestinal perforations |
| **Satralizumab**^167^ |  | 120 mg SC on weeks 0-2-4, then monthly |  | CBC with differential, liver function, infection assessment | Headache, upper respiratory tract infection, gastritis, rash, arthralgia, fatigue, nausea |
| **Secukinumab** ^168^ | Anti-IL17A mAb | 300 mg SC weekly for one month, then monthly | CBC, liver function, serologies (hepatitis, HIV, TB) | Infection assessment | Injection-side reactions, upper respiratory tract infection, inflammatory bowel disease exacerbation, depression and suicidal ideation |
| **Ixekizumab**^169^ |  | 160 mg SC, then 80 mg monthly |  |  |  |
| **Brodalumab**^170^ |  | 210 mg SC weekly for 3 weeks, then biweekly |  |  |  |
| **Belimumab**^171^ | Anti-BAFF mAb | 10 mg/kg IV on days 0, 14 y 28, then monthly  Premedication:  antihistamines +/- paracetamol | CBC, liver function, serologies (hepatitis, HIV, TB), pneumococcal vaccination | CBC with differential | Infusion reactions, leucopenia, urinary and upper respiratory tract infections, headache, depression, diarrhea, PML |
| **Blisibimod**^172^ | BAFF antagonist | 200 mg SC weekly | CBC, liver function, serologies (hepatitis, HIV, TB) | CBC, liver function | Upper respiratory and urinary tract infections, herpes zoster, injection-side reaction, diarrhea, headache, hypertension |
| **Atacicept**^89^ | Recombinant fusion protein that blocks BAFF and APRIL | 25-150 mg SC weekly | CBC, liver function, serologies (hepatitis, HIV, TB) | CBC, liver function | Injection-site reaction, upper respiratory and urinary tract infections, headache, gastrointestinal disorders |
| **Adalimumab**^90^ | Anti-TNF-α mAb | Loading dose: 160 mg SC. Day 15: 80 mg SC. From day 30, 40 mg every other week | CBC, liver function, serologies (hepatitis, HIV, TB) | CBC, liver function, infection assessment | Injection site reactions, headache, rash, malignancies, upper respiratory and urinary tract infections, demyelinating diseases, heart failure, lupus-like syndromes, cytopenias, hepatotoxicity. |
| **Certolizumab**^173^ |  | 400 mg SC on days 0, 15 and 30, then monthly |  |  |  |
| **Infliximab**^174^ |  | 5 mg/kg on days 0, 15 and 45, then every 2 months |  |  |  |
| **Etanercept**^175^ | Recombinant fusion protein that blocks circulating TNF-α | 50 mg SC weekly |  |  |  |
| **Emapalumab**^176^ | Anti-IFN-γ mAb | 1-10mg/kg IV every 3 days | CBC, liver function, serologies (hepatitis, HIV, TB) | CBC, liver function, infection assessment | Infections, hypertension, infusion reactions, pyrexia, hypokalemia, constipation, irritability, hypomagnesemia, hypoalbuminemia |
| **Effector-targeted therapies** | | | | | |
| **Drug** | **Mechanism** | **Dosage** | **Pretreatment test** | **Monitoring** | **Side effects** |
| **Efgartigimod**^125^ | IgG1 Fc-fragment to block FcRn | 10 mg/kg IV weekly for 4 weeks, then bimonthly | CBC, Ig levels, infection assessment | CBC with differential, infection assessment | Headache, upper respiratory infection, nausea, diarrhea, urinary tract infection |
| **Rozanolixizumab**^96^ | Anti-FcRn mAb | 7 mg/kg SC on days 1, 8, 15, 29, 36, and 43 |  |  | Headache, diarrhea, nausea, upper respiratory infections |
| **Nipocalimab** (NCT04951622) | Anti-FcRn mAb | 30 mg/kg IV every 2 weeks or 60mg/kg IV monthly |  |  |  |
| **Eculizumab**^98^ | Anti-C5 mAb to inhibit its cleavage into C5a and C5b | 900 mg IV weekly for the first 4 weeks, then:  • 1200 mg IV in the 5th week  • 1200 mg IV every 2 weeks | Meningococcal vaccination 2 weeks before, prophylactic antibiotic if vaccination is not possible, infection assessment | Risk of infections, especially by encapsulated bacteria such as: *Streptococcus pneumonia, Klebsiella, Haemophilus influenzae, Neisseria meningitidis, Pseudomonas aeruginosa* | Hypertension, upper respiratory tract infection, diarrhea, headache, nausea, urinary tract infection, meningococcal infections |
| **Ravulizumab**^177^ |  | Loading dose: 2400-3000 mg IV at days 0 and 15.  Maintenance: 3000-3600 mg IV every 8 weeks from day 30 |  |  |  |
| **Zilucoplan**^178^ | Synthetic macrocyclic peptide that blocks C5 | Daily 0.3 mg/kg SC | CBC, infection assessment | CBC with differential, infection assessment | Injection-site reactions, nausea, headache |
| **Therapies targeting intracellular signaling molecules** | | | | | |
| **Tolebrutinib**^179^ | BTK inhibitor | 5-60 mg PO daily | CBC, liver function, serologies (hepatitis, HIV, TB) | CBC with differential, infection assessment | Headache, upper respiratory tract infections, peripheral edemas, gastroenteritis, hepatotoxicity |
| **Evobrutinib**^109^ | BTK inhibitor | 75 mg PO daily | CBC, liver function, serologies (hepatitis, HIV, TB) |  | Upper respiratory and urinary tract infections, hepatotoxicity, lymphopenia, arthralgia, headache, flushing, malignancies |
| **Tofacitinib**^108^ | JAK1-3 inhibitor | 10-20 mg PO twice daily | CBC, liver function, serologies (hepatitis, HIV, TB) | CBC with differential, liver function, lipid profile, infection assessment  Non-melanoma skin cancer surveillance | Upper respiratory tract infection, diarrhea, headache, cytopenias, herpes zoster, hyperlipidemia, low gastrointestinal tract perforation, non-melanoma skin malignancies, hepatotoxicity |
| **Ruxolitinib**^180^ | JAK1 and 2 inhibitor | 15-20 mg PO twice daily |  |  | Cytopenias, bruising, dizziness, headache, opportunistic infections (progressive multifocal leukoencephalopathy) neuropathy |
| **Other targeted therapies** | | | | | |
| **Drug** | **Mechanism** | **Dosage** | **Pretreatment test** | **Monitoring** | **Side effects** |
| **Natalizumab**^181^ | Anti-α4 integrin mAb | 300 mg IV monthly | CBC, liver function, JCV serology, NTZ antibodies, brain MRI (JCV) | CBC with differential, liver function, JCV serology, NTZ antibodies, brain MRI every 6-12 m for JCV-seronegative patients | Infusion reactions, urinary or respiratory infections, PML (especially after 24 infusions), hepatotoxicity, herpes reactivation |
| **Abatacept**^182^ | Fusion protein that blocks CD80 | 500-750 mg IV on days 0,15 and 30, then monthly or 125 mg SC weekly | CBC, liver function, serologies (hepatitis, HIV, TB) | Infection assessment | Headache, upper respiratory tract infection, nausea |
| **Tacrolimus**^183^ | Calcineurin inhibitor | 0.3 mg/kg/day PO divided in 2 doses every 12h | CBC, liver function, serologies (hepatitis, HIV, TB) | CBC with differential, electrolytes, infection assessment | Infections, tremor, hypertension, headache, electrolyte imbalance, peripheral edemas, nephrotoxicity, hyperlipidemia, malignancies, RPLS, anemia |

Abbreviations: APRIL, proliferation-inducing ligand; BAFF, B cell-activating factor; BTK, Bruton’s tyrosine kinase; CAR-T, Chimeric Antigenic Receptor-T; CBC, complete blood count; FcRn, neonatal Fc receptor; HIV, human immunodeficiency virus; IFN, interferón; IL, interleukin; JAK, Janus kinase; JCV, John Cunningham virus; IV, intravenously; mAb, monoclonal antibody; MRI, magnetic resonance imaging; NTZ, natalizumab; PO, per oral; RPLS, Reversible Posterior Leukoencephalopathy Syndrome; SC, subcutaneous; TB, tuberculosis; TNF, tumor necrosis factor; VZV, varizela zoster virus.
